# Assessing high-throughput surrogate assays for real-world HPV16- and HPV18-specific vaccine immune monitoring: a comparison of PBNA, cCLIA, and IgG

**DOI:** 10.3389/fimmu.2026.1923707

**Published:** 2026-09-08

**Authors:** Weiya Mao, Meng Yuan, Zhaoyang Fang, Renyun Zha, Yuxiang Ye, Lin Cai, Linlin Wang, Ruopan Huang, Shuhong Luo, Cheng Guo, Conghui Liao

**Affiliations:** 1 School of Public Health, Sun Yat-Sen University, Guangzhou, China; 2 RayBiotech Co., Ltd., Guangzhou, China; 3 Department of Biomedical Engineering, School of Materials Science and Engineering, South China University of Technology, Guangzhou, China; 4 Women and Children’s Medical Center, Southern Medical University, Shenzhen, China; 5 National Medical Products Administration Key Laboratory for Quality Monitoring and Evaluation of Vaccines and Biological Products, Guangzhou, China; 6 Shenzhen Futian District Center for Disease Control and Prevention, Shenzhen, China; 7 RayBiotech Inc., Peachtree Corners, GA, United States; 8 Research Institute of Sun Yat-Sen University in Shenzhen, Shenzhen, China

**Keywords:** competitive chemiluminescent immunoassay, HPV16/18-specific antibodies, human papillomavirus vaccine, pseudovirion-based neutralization assay, real-world immune monitoring, VLP-based indirect IgG immunoassay

## Abstract

**Introduction:**

Serological monitoring is needed for the real-world evaluation of immune responses after human papillomavirus (HPV) vaccination, but the pseudovirion-based neutralization assay (PBNA) is difficult to apply at scale.

**Methods:**

This study compared PBNA, competitive chemiluminescent immunoassay (cCLIA), and a virus-like particle-based indirect IgG immunoassay for HPV16- and HPV18-specific antibody assessment. The cross-sectional component included fully vaccinated recipients and unvaccinated controls, while the longitudinal component included adolescent girls receiving a two-dose bivalent HPV vaccine. Correlation, classification performance, systematic bias, and agreement were evaluated.

**Results:**

All three assays reflected HPV16/HPV18-specific vaccine-induced antibody response trends. cCLIA showed a more stable relationship with PBNA results than the IgG assay, especially in longitudinal monitoring and after the second dose. Although the IgG assay showed lower stability than cCLIA for PBNA-defined functional classification, it consistently captured the overall pattern of vaccine-induced antibody responses at the population level. Persistent numerical differences indicated that the high-throughput assays were not directly interchangeable with neutralization titers.

**Discussion:**

These findings support a stratified strategy for HPV16- and HPV18-specific immune monitoring using PBNA as a functional laboratory reference, cCLIA for high-throughput surrogate assessment, and IgG testing for population-level trend monitoring.

## 1 Introduction

Persistent high-risk HPV infection is the primary cause of cervical cancer. It also contributes to several HPV-related anogenital and oropharyngeal diseases (1). Prophylactic HPV vaccines prevent infections and precancerous lesions caused by vaccine-targeted types. They achieve this protection by inducing type-specific antibody responses against L1 (2). With the implementation of the WHO global strategy to eliminate cervical cancer (3), HPV vaccine evaluation has expanded beyond efficacy assessment in controlled clinical trials. HPV vaccine evaluation has also expanded to post-licensure immune assessment and population-level monitoring under routine vaccination conditions. Here, “real-world immune monitoring” refers to the assessment of the magnitude, persistence, and population-level patterns of vaccine-induced antibody responses under such routine conditions outside controlled efficacy trials (4, 5). In practice, vaccine valency, vaccine product, vaccination age, and dosing schedule vary widely. Large cohorts and repeated follow-up therefore require stable and standardized serological assays. These assays support the evaluation of immune durability, immunobridging studies, and the optimization of public health immunization strategies.

Serological evaluation of HPV vaccines commonly includes three assay categories: pseudovirion-based neutralization assay (PBNA), competitive immunoassays, and virus-like particle (VLP)-based immunoglobulin G (IgG) binding antibody assays. PBNA measures the ability of serum antibodies to block HPV pseudovirion infection of target cells. By using replication-deficient pseudovirions to mimic viral entry, PBNA provides a safer laboratory surrogate for neutralization assays based on authentic virus while directly reflecting functional neutralizing activity. PBNA is widely used as a reference method for evaluating the immunogenicity of HPV VLP vaccines (6). However, PBNA requires pseudovirion preparation, live-cell culture, and strict laboratory quality control. It is technically complex, time-consuming, costly, and low-throughput (6). PBNA titers may also be affected by cell status, pseudovirion batches, and inter-laboratory variability. These limitations restrict its routine use in large-scale seroepidemiological surveys, multicenter studies, and dynamic longitudinal follow-up.

Compared with PBNA, competitive immunoassays and VLP-based IgG assays are more suitable for high-throughput testing. However, they measure different antibody properties. Competitive immunoassays detect antibodies that compete for binding to type-specific neutralization-related epitopes. They therefore provide an indirect readout of neutralization-related antibody responses (7). In contrast, VLP-based IgG assays quantify broader type-specific binding antibody responses. They can be implemented using different formats, including ELISA, multiplex IgG assays, and indirect IgG immunoassays. These high-throughput assays offer simpler workflows and greater potential for automation and standardization. However, they are not fully equivalent to PBNA because they do not directly measure functional neutralizing activity. Their interpretation, application value, and methodological boundaries in real-world settings therefore require systematic evaluation.

Previous studies have suggested that antibody levels measured by different serological assays are generally correlated after HPV vaccination. Several studies have examined the relationships between neutralization assays and other serological methods, including competitive binding assays, ELISA, and IgG assays (8, 9). These studies involved different vaccine cohorts and assay platforms (10). Overall, they reported positive correlations for HPV16- and HPV18-specific antibody responses across methods. However, most studies focused mainly on inter-assay correlation (9, 10). Correlation shows whether different assays rank individuals similarly. It does not show agreement on the original measurement scale. Therefore, correlation alone cannot support the interchangeable use of different assays at the individual level. It also cannot support threshold-based classification. In addition, the performance of alternative high-throughput assays for identifying PBNA-defined seropositivity remains insufficiently evaluated. The stability, systematic bias, and dynamic changes in inter-assay relationships also remain unclear in real-world contexts. These contexts include the parallel use of bivalent, quadrivalent, and nonavalent HPV vaccines and different stages of vaccine-induced immune responses.

HPV16 and HPV18 are the predominant high-risk HPV types responsible for cervical cancer. Together, they account for approximately 70% of cervical cancer cases worldwide (11). Type-specific antibody responses against these two types are commonly used as key endpoints in the evaluation of licensed HPV vaccines. Establishing comparability between PBNA and high-throughput assays for HPV16 and HPV18 is therefore important. It can support post-licensure evaluation of vaccine-induced immunity and the standardization of serological monitoring methods.

To address these gaps, this study assessed the comparability of PBNA, cCLIA, and a type-specific VLP-based IgG assay in measuring HPV16- and HPV18-specific antibody responses. The two high-throughput assays were selected to represent different serological readouts. These assays were evaluated for HPV16- and HPV18-specific antibody responses. A cross-sectional population of fully vaccinated individuals and unvaccinated controls was combined with a real-world longitudinal cohort of adolescents receiving a two-dose vaccination schedule. The correlations, agreement, and systematic bias among the three assays were assessed. Using PBNA as the reference method, the classification performance of cCLIA and the VLP-based indirect IgG assay for identifying seropositivity was evaluated. Furthermore, the stability and dynamic changes in inter-assay relationships were analyzed across different vaccine valencies and immune stages. This study aimed to clarify the application value and methodological boundaries of high-throughput serological assays in the evaluation of HPV16- and HPV18-specific functional neutralizing antibody responses. It also aimed to provide methodological evidence for assay selection, result comparability assessment, and standardized immune monitoring in HPV vaccine clinical trials, post-licensure real-world surveillance, and public health immunization evaluation.

## 2 Methods

### 2.1 Study design and participants

**Figure 1:**
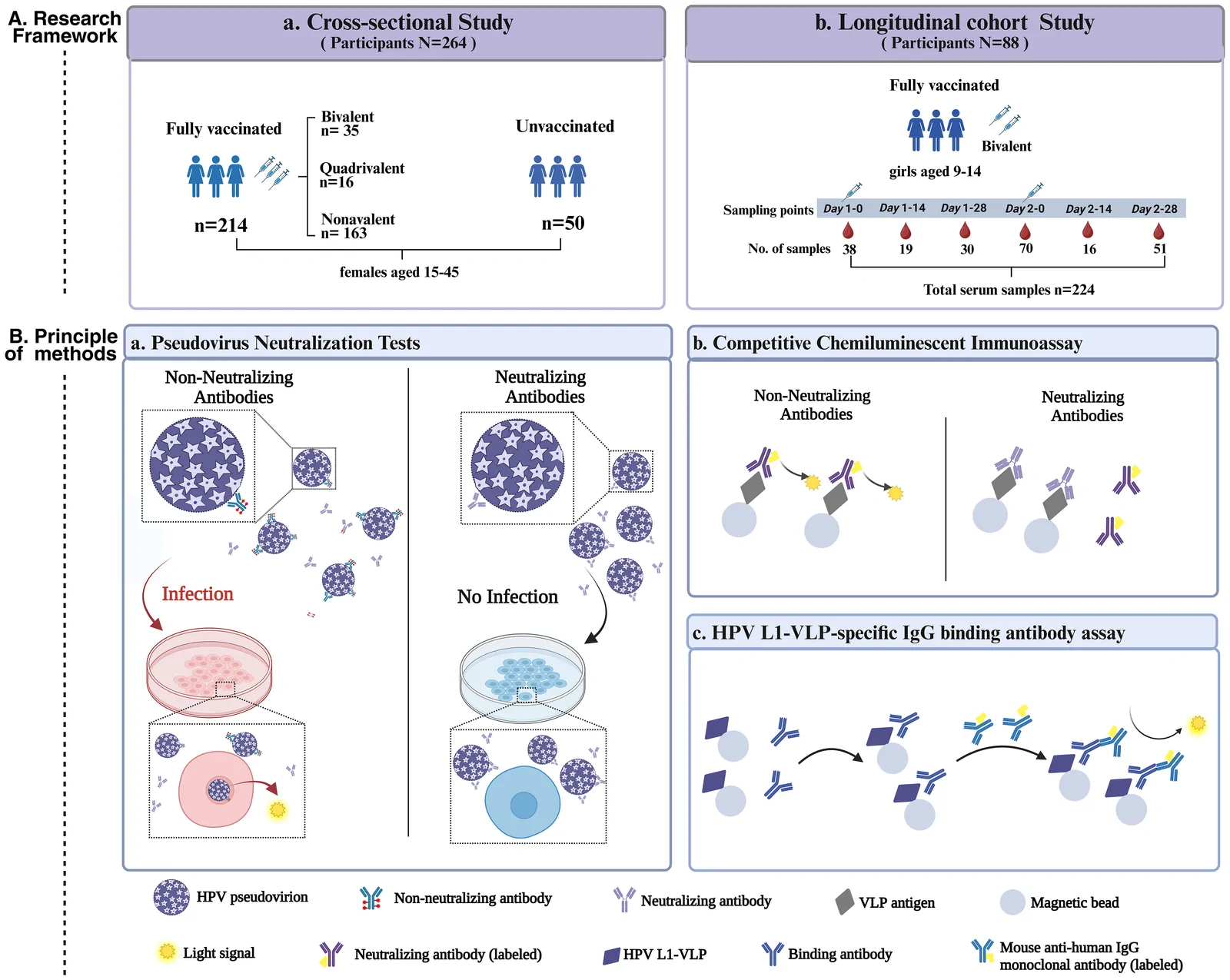
Study design and assay principles for HPV16- and HPV18-specific antibody assessment. **(A)** Overview of the study design. **(a)** The cross-sectional study included fully vaccinated females aged 15–45 years and unvaccinated controls. Fully vaccinated recipients had received bivalent, quadrivalent, or nonavalent HPV vaccines. **(b)** The longitudinal cohort included girls aged 9–14 years who received a two-dose bivalent HPV vaccination schedule, with serum samples collected at six scheduled time points. **(B)** Schematic principles of the three antibody detection methods. **(a)** PBNA measures functional neutralizing activity against HPV pseudovirions. **(b)** cCLIA measures antibodies that compete with labeled neutralizing antibodies for antigen binding. **(c)** The IgG binding antibody assay measures HPV-specific binding IgG using magnetic bead-based chemiluminescent detection. In the study-design panels, n denotes the number of participants, whereas numbers below the sampling time points denote the number of serum samples collected at each visit. HPV, human papillomavirus; PBNA, pseudovirion-based neutralization assay; cCLIA, competitive chemiluminescent immunoassay; IgG assay, IgG binding antibody assay. Created in BioRender. Conghui, L. (2026) https://BioRender.com/uzuer0i.

A combined cross-sectional and longitudinal cohort design was used in this study. The cross-sectional study was conducted in Guangdong Province from March to April 2023. A total of 214 females aged 15–45 years who had completed a full HPV vaccination series and 50 unvaccinated controls were included (**Figures 1A, a**). Vaccination records of the vaccinated participants were retrieved from the Guangdong Provincial Immunization Information System. The administered vaccines included the domestic bivalent HPV vaccine (Cecolin), imported bivalent HPV vaccine (Cervarix), imported quadrivalent HPV vaccine (Gardasil), and imported nonavalent HPV vaccine (Gardasil 9). Participants who were pregnant or receiving immune-modulating treatment were excluded.

The longitudinal cohort included 88 adolescent girls aged 9–14 years from Baiyun District, Guangzhou (**Figures 1A, b**). All participants received the domestic bivalent HPV vaccine (Cecolin) according to a two-dose schedule, with an interval of approximately 6 months between doses. Solicited local reactogenicity and systemic adverse events were actively monitored and recorded after each vaccination during follow-up. Participants were excluded if they deviated from the scheduled vaccination program, had severe metabolic or malignant diseases, were receiving systemic immunosuppressive therapy, were HIV-positive, or experienced serious vaccination-related adverse events. Peripheral venous blood samples (5 mL) were collected before the first dose, 14 and 28 days after the first dose, immediately before the second dose, and 14 and 28 days after the second dose.

Serum samples were separated by centrifugation, aliquoted, and stored at −80 °C until testing. This study was approved by the Ethics Committee of the School of Public Health, Sun Yat-sen University. Written informed consent was obtained from all participants or their legal guardians before enrollment.

### 2.2 Serological assays

#### 2.2.1 Pseudovirion-based neutralization assay

HPV16- and HPV18-specific neutralizing antibody titers were measured by PBNA (**Figures 1B, a**). HPV pseudovirions were produced by co-transfecting HEK293T cells (Procell, China) with HPV16 or HPV18 structural gene plasmids (Addgene, USA) and a fluorescent reporter plasmid (Addgene, USA). After amplification, pseudovirion titers were determined by infection assays using 293FT cells (Procell, China).

For the neutralization assay, test sera were serially diluted fivefold from 1:40 to 1:125,000. Diluted sera were mixed with a fixed titer of pseudovirions and incubated at 4 °C for 1 h. The mixtures were then added to pre-seeded 293FT cells and cultured for 72 h. After incubation, the supernatant was discarded, and cells were lysed with lysis buffer. Fluorescent spot counts in each well were measured using an enzyme-linked immunospot reader (Cellular Technology Ltd., USA). The inhibition rate at each serum dilution was calculated using pseudovirion control wells without serum neutralization as the reference. The serum dilution corresponding to 50% inhibition was calculated using the Reed–Muench method and was defined as the neutralizing antibody titer ID_50_. The Reed–Muench calculation estimates the 50% endpoint from the serial-dilution response rather than reporting the tested fivefold dilution levels directly; therefore, the resulting ID_50_ values were not restricted to discrete dilution endpoints. Four-parameter logistic curve fitting was not used for PBNA. Positive and negative reference sera were included in each run to monitor within-run and between-run variation. PBNA seropositivity was defined as ID_50_ > 40 for both HPV16 and HPV18; values ≤40 were classified as PBNA-negative for assay-based serostatus assessment and were not interpreted as confirmed biological non-response.

#### 2.2.2 Competitive antibody assay

HPV16- and HPV18-specific competitive binding antibody levels were measured by cCLIA (**Figures 1B, b**). Magnetic beads coated with HPV16/18 L1-VLP antigens (Wantai BioPharm, China) were used as the solid phase. Acridinium ester-labeled type-specific monoclonal antibodies were used as competing detection antibodies. Competitive antibody levels were quantified by measuring the competition between serum antibodies and labeled monoclonal antibodies for antigen-binding sites.

The assay was performed on an automated chemiluminescence immunoassay analyzer (SMART-6500, Chongqing Cosmo Biotechnology Co., Ltd., China). Serum samples, labeled monoclonal antibodies, and antigen-coated magnetic beads were added to reaction cuvettes. After incubation at 37 °C for 20 min, magnetic separation and washing were performed to remove unbound materials. Chemiluminescent substrate solution was then added, and relative light units (RLUs) were measured. Antibody concentrations were calculated from serial calibrators using a four-parameter logistic curve. Positive and negative quality controls were included in each run to monitor within-run and between-run variation. The analytical measuring range was 11.0–400 IU/L for HPV16 and 1.5–1000 IU/L for HPV18. The manufacturer-recommended positivity threshold was >5 IU/L for both HPV16 and HPV18.

#### 2.2.3 IgG binding antibody assay

HPV16- and HPV18-specific IgG binding antibody levels were quantified by the IgG assay (**Figures 1B, c**). Magnetic beads coated with HPV16/18 L1-VLP antigens (Wantai BioPharm, China) were used as the solid phase. Acridinium ester-labeled mouse anti-human IgG monoclonal antibodies were used as detection antibodies. Bound serum IgG antibodies were detected through chemiluminescent signal generation.

The assay was performed on an automated chemiluminescence immunoassay analyzer (SMART-6500, Chongqing Cosmo Biotechnology Co., Ltd., China). Serum samples and antigen-coated magnetic beads were incubated at 37 °C for 12 min. After magnetic separation and washing, acridinium ester-labeled mouse anti-human IgG antibodies were added and incubated at 37 °C for 8 min. A second magnetic separation and washing step was then performed. Chemiluminescent substrate solution was added, and RLUs were measured. Antibody concentrations were calculated from serial calibrators using a four-parameter logistic curve. Positive and negative quality controls were included in each run to monitor assay accuracy. The analytical measuring range was 2–1000 IU/L for HPV16 and 2–3000 IU/L for HPV18. The manufacturer-recommended positivity threshold was >10 IU/L for both HPV16 and HPV18.

### 2.3 Statistical analysis

#### 2.3.1 Data preprocessing and descriptive analysis

All statistical analyses were performed using R software version 4.2.0. Continuous variables were summarized as medians and interquartile ranges according to their distributions. Categorical variables were summarized as frequencies and percentages. In the cross-sectional study, differences among vaccine-type groups were assessed using the Kruskal–Wallis test, chi-square test, or Fisher’s exact test, as appropriate. PBNA results were summarized as geometric mean titers (GMTs), whereas cCLIA and IgG results were summarized as geometric mean concentrations (GMCs). Corresponding 95% confidence intervals (CIs) were calculated.

For continuous numerical analyses, PBNA results below the lower reporting boundary (<40) were assigned a value of 20, whereas values above the upper limit of detection of 125,000 were assigned a value of 125,000. Blank, non-numeric, missing, and non-finite values in cCLIA and IgG results were coded as missing. All valid raw values were log_10_-transformed when they were greater than 0 and finite. Pairwise complete observations were used for all pairwise assay comparisons. The assigned PBNA value of 20 was used only for continuous numerical analyses and was not interpreted as the true neutralizing antibody titer. Qualitative classification agreement and receiver operating characteristic (ROC) analyses were still based on PBNA seropositivity defined as ID50 > 40. Unvaccinated controls were used only to describe background antibody distributions and to extend the evaluation of qualitative classification performance. They were not included in the primary continuous analyses of correlation, agreement, or systematic bias.

#### 2.3.2 Quantitative methodological comparison

Continuous numerical relationships among the three assays were evaluated using Spearman’s rank correlation coefficient, intraclass correlation coefficient (ICC), and Bland–Altman analysis. Spearman’s correlation coefficient was used to assess monotonic associations between log10-transformed values. Because PBNA, cCLIA, and IgG differed in assay principle, measurement unit, and numerical range, scale-adjusted relative consistency was evaluated using ICCs based on Z-score-standardized log_10_ values. Z-score standardization was used to place the three assays on a common standardized scale while retaining their relative variation. Absolute numerical agreement was evaluated using absolute-agreement ICCs based on unstandardized log_10_-transformed values. Because the same predefined assay platforms were applied to each sample and the analysis focused on individual assay measurements, two-way single-measure ICC models were used. Scale-adjusted relative consistency was assessed using a two-way consistency, single-measure ICC [ICC(C,1)] based on Z-score-standardized log_10_ values. Absolute numerical agreement was assessed using a two-way absolute-agreement, single-measure ICC [ICC(A,1)] based on unstandardized log_10_ values.

Bland–Altman analysis was used to assess systematic bias and individual-level differences between each alternative assay and PBNA. The analysis was performed using log_10_-transformed values. Difference directions were defined as log_10_(PBNA) − log_10_ (cCLIA), log_10_ (PBNA) − log_10_ (IgG), or log_10_ (cCLIA) − log_10_ (IgG), as appropriate. Mean bias, 95% limits of agreement (LoA), and the width of the LoA were calculated. Proportional bias was formally assessed by regressing paired differences on the corresponding paired means. For longitudinal proportional-bias analyses, participant-clustered HC1 robust standard errors were used to account for repeated measurements. As a sensitivity analysis, Bland–Altman, Spearman correlation, consistency ICC, and absolute-agreement ICC analyses were repeated after excluding PBNA values below the reporting threshold. To further assess the potential influence of within-participant dependence in the longitudinal cohort, Spearman correlation and Bland–Altman analyses were additionally repeated using participant-level mean log_10_ values calculated across available longitudinal time points.

Exploratory inter-assay calibration was further performed using Passing–Bablok regression, with log_10_-transformed PBNA ID_50_ as the dependent variable and log_10_-transformed cCLIA or IgG concentrations as predictors. Separate models were fitted for HPV16 and HPV18 in the cross-sectional vaccinated samples and longitudinal cohort. Baseline samples were excluded from the longitudinal calibration models. Ninety-five percent confidence intervals were estimated using 2,000 bootstrap resamples, with participant-level cluster bootstrap used for the longitudinal cohort to account for repeated measurements. Calibration equations were reported on both the log_10_ and back-transformed scales.

#### 2.3.3 Qualitative classification agreement and ROC analysis

PBNA-defined neutralizing antibody status was used as the functional reference standard to evaluate the classification performance of cCLIA and IgG. cCLIA > 5 IU/L and IgG > 10 IU/L were defined as positive results for the alternative assays. Sensitivity, specificity, positive predictive value (PPV), negative predictive value (NPV), overall percent agreement, Youden index, and Cohen’s kappa were calculated from 2 × 2 contingency tables.

ROC curves were generated using the raw continuous values of the alternative assays as predictors and PBNA-defined seropositivity as the reference outcome. The 95% CI of the area under the curve (AUC) was estimated using the DeLong method. The optimal cut-off value was determined by maximizing the Youden index.

Because PBNA-negative samples were limited among fully vaccinated participants in the cross-sectional study, and all HPV16 PBNA results were positive, the vaccinated-only analysis was mainly used to assess positive agreement. To ensure sufficient PBNA-negative samples, the primary ROC/AUC analysis was performed using the combined dataset of fully vaccinated participants and unvaccinated controls. This combined analysis was used to evaluate the overall ability of the alternative assays to distinguish PBNA-positive from PBNA-negative samples. It was not used as the primary basis for assessing continuous numerical relationships between the alternative assays and PBNA within vaccinated participants.

In the combined cross-sectional dataset of vaccinated recipients and unvaccinated controls, exploratory combined-classification strategies using cCLIA and IgG were further evaluated for PBNA-defined seropositivity. Three predefined rules were assessed using the manufacturer-recommended cut-offs: a parallel OR rule, in which samples positive by either cCLIA or IgG were classified as positive; a serial AND rule, in which only samples positive by both assays were classified as positive; and an IgG-first sequential rule, in which IgG was used as the first-line screening assay and IgG-positive samples were further assessed by cCLIA.

#### 2.3.4 Stratified and longitudinal analyses

To evaluate the stability of the relationships between the alternative assays and PBNA across vaccine types, vaccinated samples from the cross-sectional study were stratified by vaccine type, including bivalent, quadrivalent, and nonavalent HPV vaccines. Because the quadrivalent vaccine group had a small sample size, its stratified results were interpreted with caution. As a product-level sensitivity analysis, the bivalent group was further separated into Cecolin and Cervarix recipients, and product-specific analyses were performed across Cecolin, Cervarix, Gardasil, and Gardasil 9 recipients. Product-stratified scatter plots were generated, and Spearman correlation coefficients, consistency ICCs, Bland–Altman bias, and absolute-agreement ICCs were estimated separately for each vaccine product.

Because age and time since the last vaccine dose differed across vaccine groups, multivariable linear regression was additionally used to assess their potential influence on inter-assay differences. Separate models were fitted for each HPV type and PBNA–surrogate assay comparison. Inter-assay differences were defined as log_10_ (PBNA) − log_10_ (cCLIA) or log_10 (_PBNA) − log_10_ (IgG), with vaccine product, age, and time since the last vaccination included simultaneously as predictors. Vaccine product was treated as a categorical variable, and time since vaccination was scaled per 100 days. Product-level analyses were considered exploratory because of the relatively small sample sizes of some vaccine-product subgroups.

In the longitudinal cohort, linear mixed-effects models were used to evaluate changes in HPV16- and HPV18-specific antibody levels over time after vaccination. Log_10_-transformed antibody levels were used as dependent variables. Sampling time point was included as a fixed effect, and participant was included as a random intercept. Pairwise comparisons between time points were adjusted for multiple testing using the Bonferroni method.

We further stratified the longitudinal cohort by post-vaccination follow-up time point. This analysis examined whether the relationships between cCLIA, the IgG assay, and PBNA changed across vaccination stages. Baseline samples were included in the pooled longitudinal correlation, agreement, and classification analyses, but were excluded from post-vaccination time-point-specific agreement analyses because antibody levels were close to the lower measurement range for most assays. Baseline samples were also excluded from the longitudinal calibration models, as described above. Samples collected 14 and 28 days after the first dose and 14 days after the second dose were defined as dynamic antibody response phases. Samples collected immediately before the second dose and 28 days after the second dose were designated as relatively stable plateau phases.

## 3 Results

### 3.1 Overview of study participants and serum samples

After review of information completeness and sample availability, a total of 352 participants were included in the study framework. The cross-sectional study included 264 participants, comprising 214 fully vaccinated HPV vaccine recipients and 50 unvaccinated controls. The longitudinal cohort included 88 adolescent girls who received a two-dose bivalent HPV vaccine. The composition of the study population and the flow of serum samples are summarized in the research framework (**Figure 1A**).

Among the cross-sectional vaccinated recipients, 214 serum samples were available for serological analysis. In the longitudinal cohort, 224 serum samples were collected across six scheduled sampling time points. The median age was 25.0 years among cross-sectional vaccinated recipients and 13.0 years among longitudinal cohort participants. Median height was 162.0 cm and 158.0 cm, respectively, and median BMI was 20.26 kg/m² and 18.8 kg/m², respectively. Because the cross-sectional vaccinated recipients and longitudinal cohort participants represented different study designs and age groups, no formal statistical comparisons were performed between these two populations. The characteristics of the vaccinated participants and serum samples are summarized (**Table 1**).

**Table 1:** Overall characteristics of study participants and serum samples.

Characteristic	Cross-sectional vaccinated recipients	Longitudinal cohort
Participants, n	214	88
Serum samples, n	214	224
Age, years, median (IQR)	25.0 (24.0–26.0)	13.0 (12.0-13.0)
Height, cm, median (IQR)	162.0 (158.0–166.0)	158.0 (155.0-162.0)
BMI, kg/m², median (IQR)	20.26 (19.03–21.56)	18.8 (17.5-20.5)

In addition, 50 unvaccinated controls with 50 corresponding serum samples were included in the cross-sectional study. These samples were used as a negative-background reference group and contributed to subsequent classification performance analyses. Therefore, their demographic characteristics were not further summarized in **Table 1**.

### 3.2 Overall correlations among antibody levels measured by the three assays

**Figure 2:**
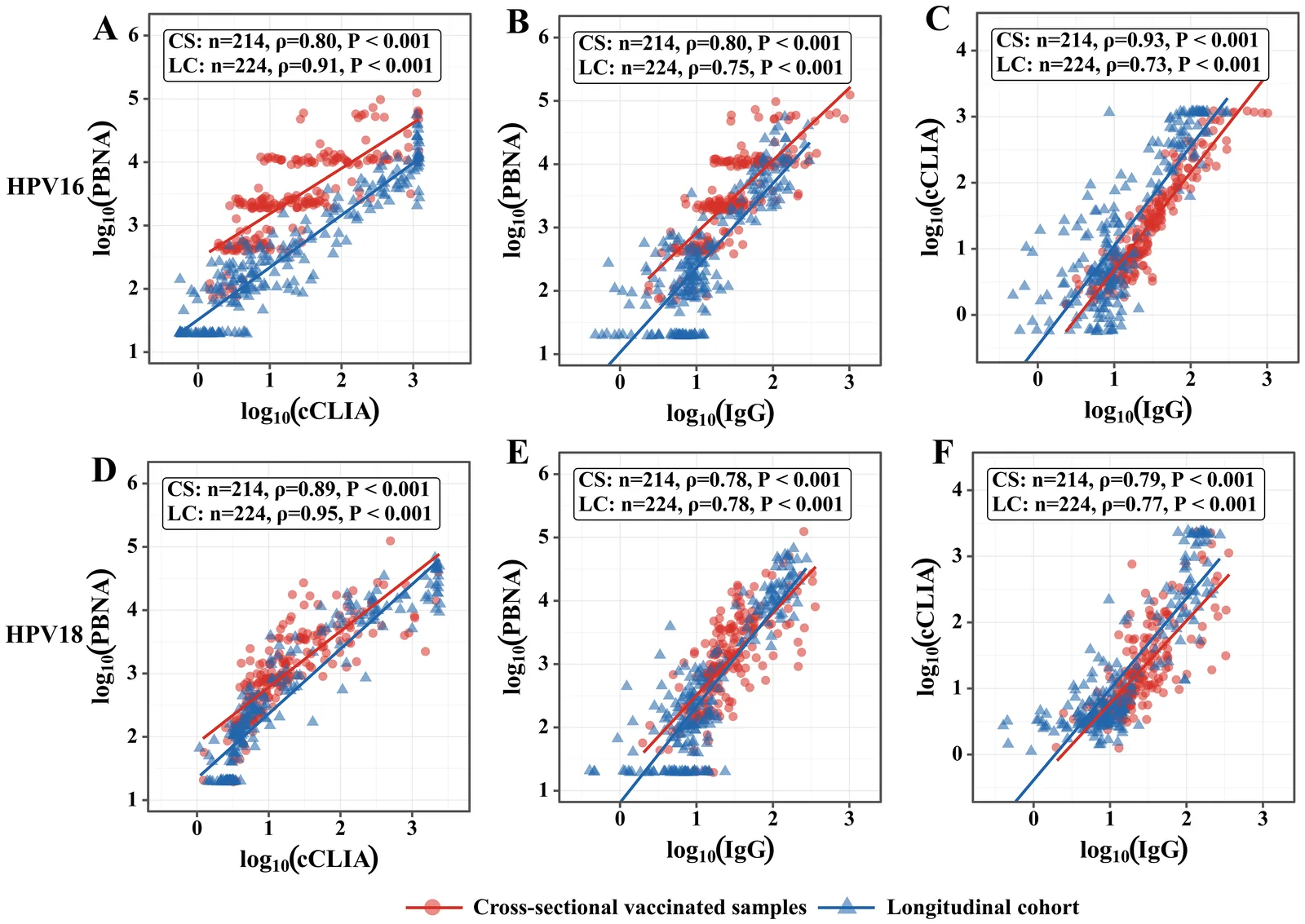
Correlations among HPV16- and HPV18-specific antibody levels measured by PBNA, cCLIA, and IgG binding antibody assays. **(A)** Correlation between HPV16-specific PBNA and cCLIA results. **(B)** Correlation between HPV16-specific PBNA and IgG results. **(C)** Correlation between HPV16-specific cCLIA and IgG results. **(D)** Correlation between HPV18-specific PBNA and cCLIA results. **(E)** Correlation between HPV18-specific PBNA and IgG results. **(F)** Correlation between HPV18-specific cCLIA and IgG results. Red circles indicate cross-sectional vaccinated samples, and blue triangles indicate pooled longitudinal cohort serum samples. All assay values were log_10_-transformed before analysis. Spearman’s rank correlation coefficients and P values were calculated separately for the two study populations using pairwise complete observations. Fitted lines are shown for trend visualization only. PBNA values are expressed as ID_50_, whereas cCLIA and IgG values are expressed in IU/L. In each panel, n denotes the number of serum samples included in the corresponding analysis. CS, cross-sectional vaccinated samples; LC, pooled longitudinal cohort serum samples; ρ, Spearman’s rank correlation coefficient; PBNA, pseudovirion-based neutralization assay; cCLIA, competitive chemiluminescent immunoassay; IgG assay, IgG binding antibody assay.

Positive pairwise correlations were observed among PBNA, cCLIA, and IgG results for both HPV16 and HPV18, indicating that the three assays generally preserved similar relative rankings of vaccine-induced antibody levels (**Figure 2**). For HPV16, correlations in the cross-sectional vaccinated samples were high for PBNA versus cCLIA (ρ = 0.80), PBNA versus IgG (ρ = 0.80), and cCLIA versus IgG (ρ = 0.93). In the pooled longitudinal serum samples, the PBNA–cCLIA correlation was stronger (ρ = 0.91), whereas the PBNA–IgG and cCLIA–IgG correlations were moderate to high, with ρ values of 0.75 and 0.73, respectively (**Figures 2A–C**). For HPV18, PBNA showed high correlations with cCLIA in both the cross-sectional vaccinated samples and pooled longitudinal serum samples (ρ = 0.89 and 0.95, respectively). Correlations between PBNA and IgG and between cCLIA and IgG were also positive in both study populations, with ρ values ranging from 0.77 to 0.79 (**Figures 2D–F**). All pairwise correlations were statistically significant (*P* < 0.001).

The horizontal clustering was localized to the HPV16 PBNA measurements, as similar banding was observed in both PBNA–cCLIA and PBNA–IgG plots but not in the cCLIA–IgG comparison. Re-examination of the underlying PBNA values showed that this pattern did not reflect discrete endpoint reporting or widespread upper-limit saturation. Among the 214 samples, 198 distinct HPV16 ID_50_ values were observed, and only one sample reached the highest tested dilution of 125,000. The clustering was also not confined to bivalent vaccine recipients. Thus, the apparent banding is consistent with concentration of continuously estimated Reed–Muench endpoints within particular titer intervals generated by the fivefold serial-dilution scheme, rather than true discrete quantization.

Sensitivity analyses supported the robustness of these correlations. Participant-level mean analyses produced similar correlation patterns (**Supplementary Figure 1**), and exclusion of PBNA values below the reporting boundary resulted in only small and mixed changes in Spearman coefficients (all |Δρ| < 0.029; **Supplementary Table 1**).

### 3.3 Classification performance of cCLIA and IgG for PBNA-defined seropositivity

**Figure 3:**
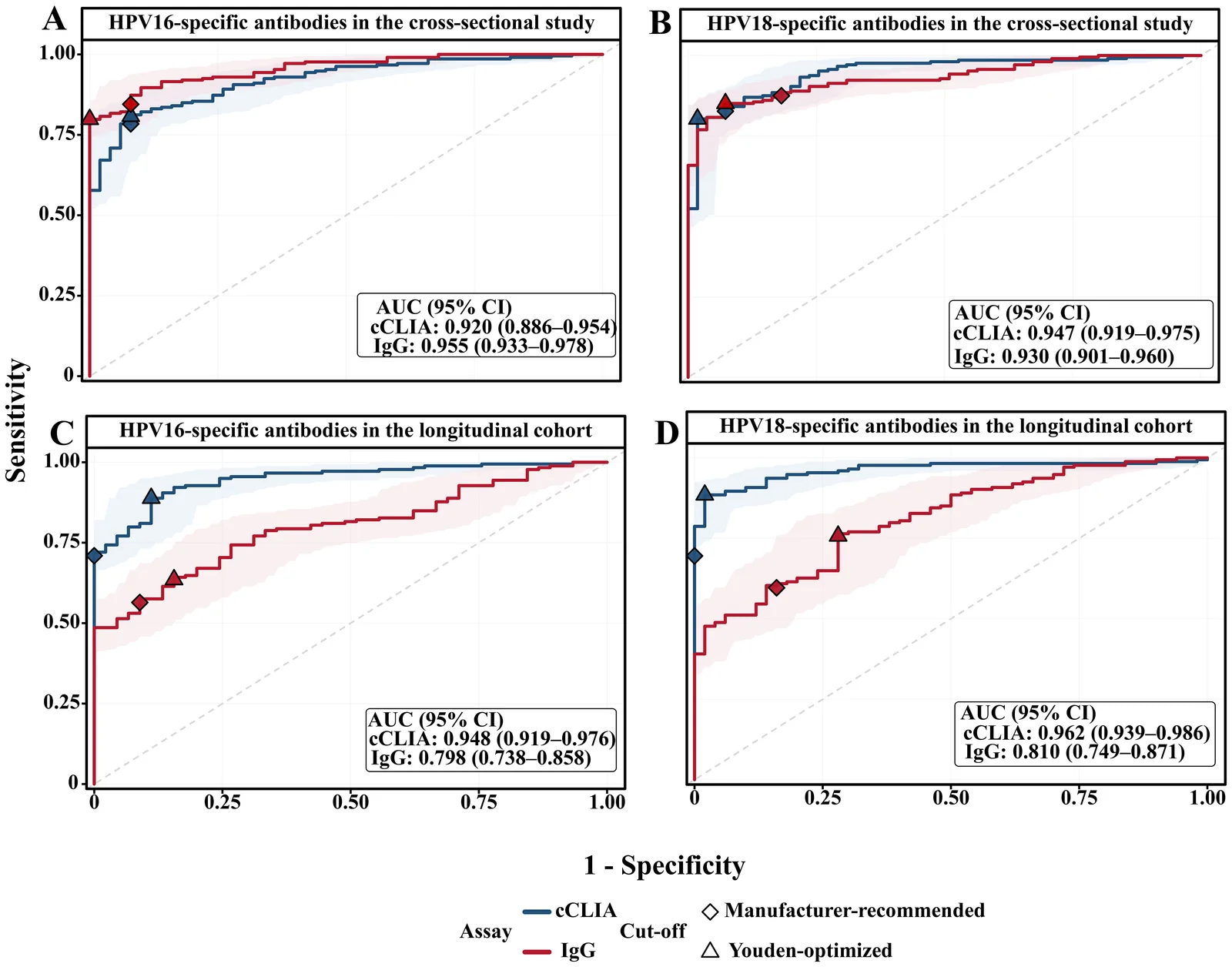
Classification performance of cCLIA and IgG for PBNA-defined HPV16- and HPV18-specific seropositivity. **(A)** ROC curves for HPV16-specific antibodies in the cross-sectional study. **(B)** ROC curves for HPV18-specific antibodies in the cross-sectional study. **(C)** ROC curves for HPV16-specific antibodies in the longitudinal cohort. **(D)** ROC curves for HPV18-specific antibodies in the longitudinal cohort. Blue curves indicate cCLIA, and red curves indicate IgG. Shaded areas represent 95% confidence intervals. Diamond symbols indicate manufacturer-recommended cut-offs, and triangle symbols indicate Youden-optimized cut-offs. The diagonal dashed line represents the no-discrimination reference line. PBNA-defined seropositivity, defined as ID_50_ > 40, was used as the functional reference assay. AUC values with 95% confidence intervals are shown in each panel. ROC, receiver operating characteristic; AUC, area under the ROC curve; CI, confidence interval; PBNA, pseudovirion-based neutralization assay; cCLIA, competitive chemiluminescent immunoassay; IgG assay, IgG binding antibody assay.

To evaluate the ability of high-throughput assays to classify PBNA-defined seropositivity, ROC curve analyses were performed for cCLIA and IgG in both the cross-sectional study and the longitudinal cohort. PBNA seropositivity, defined as ID_50_ > 40, was used as the reference standard (**Figure 3**). Detailed threshold-based classification metrics for the cross-sectional study and the longitudinal cohort are presented in **Supplementary Tables 2A, B** respectively.

In the cross-sectional study, both assays showed good discriminatory performance. For HPV16-specific antibodies, the AUC was 0.955 (95% CI: 0.933–0.978) for IgG and 0.920 (95% CI: 0.886–0.954) for cCLIA (**Figure 3A**). For HPV18-specific antibodies, the AUC was 0.947 (95% CI: 0.919–0.975) for cCLIA and 0.930 (95% CI: 0.901–0.960) for IgG (**Figure 3B**). Threshold-based analyses using both manufacturer-recommended and Youden-optimized cut-offs showed moderate to substantial agreement for both assays. These results indicated that both cCLIA and IgG were able to discriminate PBNA-positive from PBNA-negative samples in the cross-sectional setting. Detailed sensitivity, specificity, Youden index, Kappa values, and contingency table results are presented in **Supplementary Table 2A**.

In the combined cross-sectional dataset of vaccinated recipients and unvaccinated controls, the two high-throughput assays showed complementary classification properties for PBNA-defined seropositivity (**Supplementary Table 3**). IgG alone provided relatively high sensitivity, whereas cCLIA alone provided relatively high specificity. When the two assays were combined, the parallel OR rule prioritized sensitivity, reaching 87.9% for HPV16 and 91.9% for HPV18. In contrast, the serial AND rule maximized specificity, which reached 100.0% for both HPV16 and HPV18. For HPV18, the AND rule also achieved 100.0% positive predictive value. The IgG-first sequential rule produced the same final classification as the AND rule, but required cCLIA testing only for IgG-positive samples, reducing the cCLIA testing proportion to 70.1% for HPV16 and 73.1% for HPV18. These findings suggest that combined interpretation may be used to tailor high-throughput classification strategies according to different monitoring objectives, including sensitivity prioritization, specificity prioritization, or reduction of confirmatory testing burden.

In the longitudinal cohort, different performance patterns were observed between cCLIA and IgG. cCLIA maintained high discriminatory performance for PBNA-defined seropositivity, with AUCs of 0.948 (95% CI: 0.919–0.976) for HPV16 and 0.962 (95% CI: 0.939–0.986) for HPV18 (**Figures 3C, D**). In contrast, IgG showed lower AUCs in the longitudinal cohort, with values of 0.798 (95% CI: 0.738–0.858) for HPV16 and 0.810 (95% CI: 0.749–0.871) for HPV18 (**Figures 3C, D**).

Threshold-based agreement analyses were consistent with the ROC findings. After Youden index optimization, cCLIA achieved a better balance between sensitivity and specificity, with Kappa values of 0.691 for HPV16 and 0.762 for HPV18 (**Supplementary Table 2B**). By contrast, the corresponding Kappa values for IgG were 0.330 and 0.406, respectively.

After Youden index optimization, cCLIA sensitivities increased to 88.8% and 88.5%, with kappa values of 0.691 and 0.762, whereas IgG sensitivities were 64.2% and 76.4%, with kappa values of 0.330 and 0.406. These findings indicate that fixed manufacturer-recommended IgG thresholds may miss a substantial proportion of PBNA-positive samples in longitudinal monitoring. At the manufacturer-recommended cut-offs, cCLIA showed sensitivities of 70.9% for HPV16 and 69.5% for HPV18, with corresponding kappa values of 0.495 and 0.505. The corresponding sensitivities of the IgG assay were lower, at 56.4% and 60.3%, with kappa values of 0.294 and 0.309, respectively (**Supplementary Table 2B**). Overall, both cCLIA and IgG showed good discriminatory performance for PBNA-defined seropositivity in the cross-sectional study. In the longitudinal cohort, however, cCLIA showed more stable classification performance than IgG, particularly when threshold-based agreement was considered.

### 3.4 Absolute agreement and systematic bias across assays

**Figure 4:**
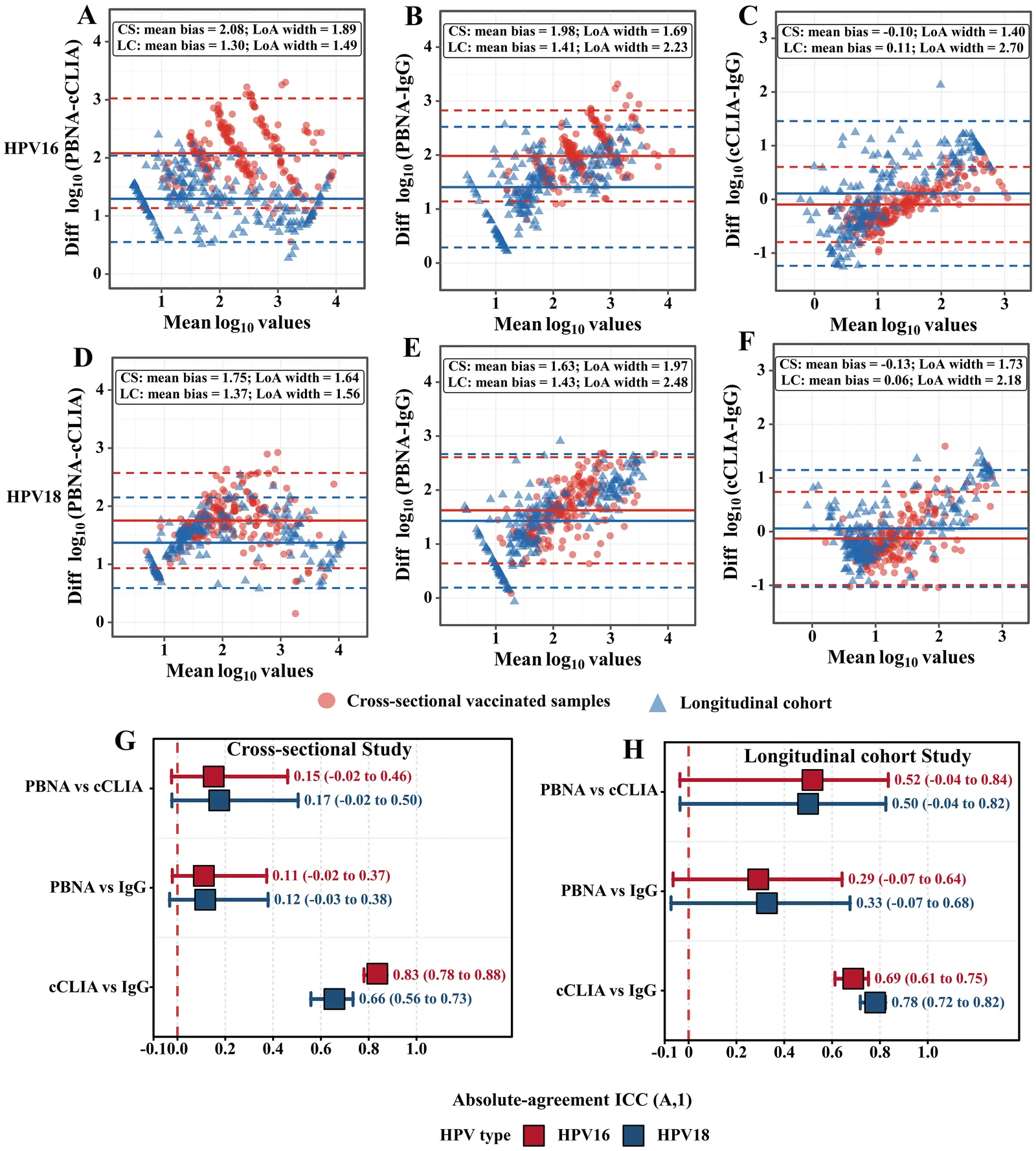
Quantitative agreement and systematic bias among PBNA, cCLIA, and IgG assays. **(A)** Bland–Altman plot for HPV16-specific PBNA versus cCLIA. **(B)** Bland–Altman plot for HPV16-specific PBNA versus IgG. **(C)** Bland–Altman plot for HPV16-specific cCLIA versus IgG. **(D)** Bland–Altman plot for HPV18-specific PBNA versus cCLIA. **(E)** Bland–Altman plot for HPV18-specific PBNA versus IgG. **(F)** Bland–Altman plot for HPV18-specific cCLIA versus IgG. **(G)** Absolute-agreement ICCs in the cross-sectional vaccinated samples. **(H)** Absolute-agreement ICCs in the longitudinal cohort. In panels **(A–F)**, the x-axis represents the mean of two log_10_-transformed assay values, and the y-axis represents their difference. Difference directions are defined as PBNA minus cCLIA, PBNA minus IgG, or cCLIA minus IgG. Solid horizontal lines indicate mean bias, and dashed horizontal lines indicate the 95% limits of agreement. Red circles indicate cross-sectional vaccinated samples, and blue triangles indicate longitudinal cohort serum samples. In panels **(G, H)**, squares indicate absolute-agreement ICC estimates, and horizontal bars indicate the corresponding 95% confidence intervals. Red and blue squares represent HPV16 and HPV18, respectively. PBNA results are expressed as ID_50_, whereas cCLIA and IgG results are expressed in IU/L. All analyses were performed using log_10_-transformed values. CS, cross-sectional vaccinated samples; LC, longitudinal cohort serum samples; ICC, intraclass correlation coefficient; LoA, limits of agreement; PBNA, pseudovirion-based neutralization assay; cCLIA, competitive chemiluminescent immunoassay; IgG assay, IgG binding antibody assay.

Although the three assays showed consistent correlation patterns and the high-throughput assays demonstrated discriminatory performance for PBNA-defined seropositivity, their absolute agreement on the continuous scale required further evaluation. Bland–Altman analysis and absolute-agreement ICCs were therefore used to assess systematic bias and numerical agreement across assays (**Figure 4**).

Bland–Altman analysis showed persistent positive systematic bias between PBNA and the two high-throughput assays (**Figures 4A, B, D, E**). For HPV16 and HPV18, the mean bias ranged from 1.30 to 2.08 for PBNA versus cCLIA and from 1.41 to 1.98 for PBNA versus IgG. This bias was observed in both the cross-sectional vaccinated samples and the longitudinal cohort, indicating clear differences in numerical scale between PBNA and either high-throughput assay. By contrast, the mean bias between cCLIA and IgG was close to zero in both study populations, although the limits of agreement remained relatively wide, especially in the longitudinal cohort (**Figures 4C, F**).

The absolute-agreement ICCs showed a similar pattern. Agreement between PBNA and cCLIA or IgG was limited on the log_10_-transformed numerical scale. This was most evident in the cross-sectional vaccinated samples, where the ICCs were 0.15–0.17 for PBNA versus cCLIA and 0.11–0.12 for PBNA versus IgG (**Figure 4G**). In the longitudinal cohort, the ICCs for PBNA versus cCLIA increased to 0.50–0.52, whereas those for PBNA versus IgG remained lower, at 0.29–0.33 (**Figure 4H**). In comparison, cCLIA and IgG showed higher ICCs across both study populations, suggesting more similar numerical scales between the two high-throughput assays.

To account for the repeated-measures structure of the longitudinal cohort, a subject-mean Bland–Altman sensitivity analysis was also performed. After participant-level mean values were calculated from log_10_-transformed assay results across all available sampling time points, similar bias patterns were observed. For HPV16, the mean biases for PBNA versus cCLIA, PBNA versus IgG, and cCLIA versus IgG were 1.32, 1.38, and 0.06, respectively. For HPV18, the corresponding mean biases were 1.38, 1.41, and −0.03, respectively (**Supplementary Figure 2**). These results were generally consistent with the sample-level analysis of the longitudinal cohort.

Sensitivity analyses further supported the main agreement findings. Exclusion of PBNA values below the reporting boundary resulted in only small and mixed changes in Spearman correlations (all |Δρ| < 0.029) and consistency ICCs (maximum |ΔICC| = 0.027). The influence on proportional bias was comparison dependent. In the longitudinal cohort, the HPV18 PBNA–cCLIA slope changed from positive in the full analysis (β = 0.096, 95% CI: 0.035–0.156; *P* = 0.002) to modestly negative after exclusion of PBNA ID_50_ <40 observations (β = −0.057, 95% CI: −0.113 to −0.0003; *P* = 0.049), whereas significant positive proportional bias persisted for PBNA–IgG for both HPV16 and HPV18. Absolute-agreement ICCs did not improve after restriction and decreased in all four longitudinal comparisons, with a maximum absolute change of 0.070 (**Supplementary Figure 3**; **Supplementary Table 4**). These findings indicate that lower-bound handling influenced selected agreement estimates but did not fully explain the observed inter-assay differences. Given the persistent numerical differences across assays, exploratory Passing–Bablok regression yielded assay-specific calibration equations for estimating PBNA ID_50_ from cCLIA or IgG measurements (**Supplementary Table 5**). The calibration coefficients differed across HPV types and study populations, indicating that a single universal conversion equation was not supported.

### 3.5 Stability of assay relationships across vaccine types and vaccination products

To further evaluate whether the relationships of PBNA with cCLIA and IgG were stable in different application settings, stratified analyses were performed from two perspectives. In the cross-sectional vaccinated samples, analyses were stratified by vaccine type to assess whether the assay relationships were consistent across vaccine backgrounds. The basic characteristics of vaccinated participants by vaccine type are shown in **Supplementary Table 6**.

**Figure 5:**
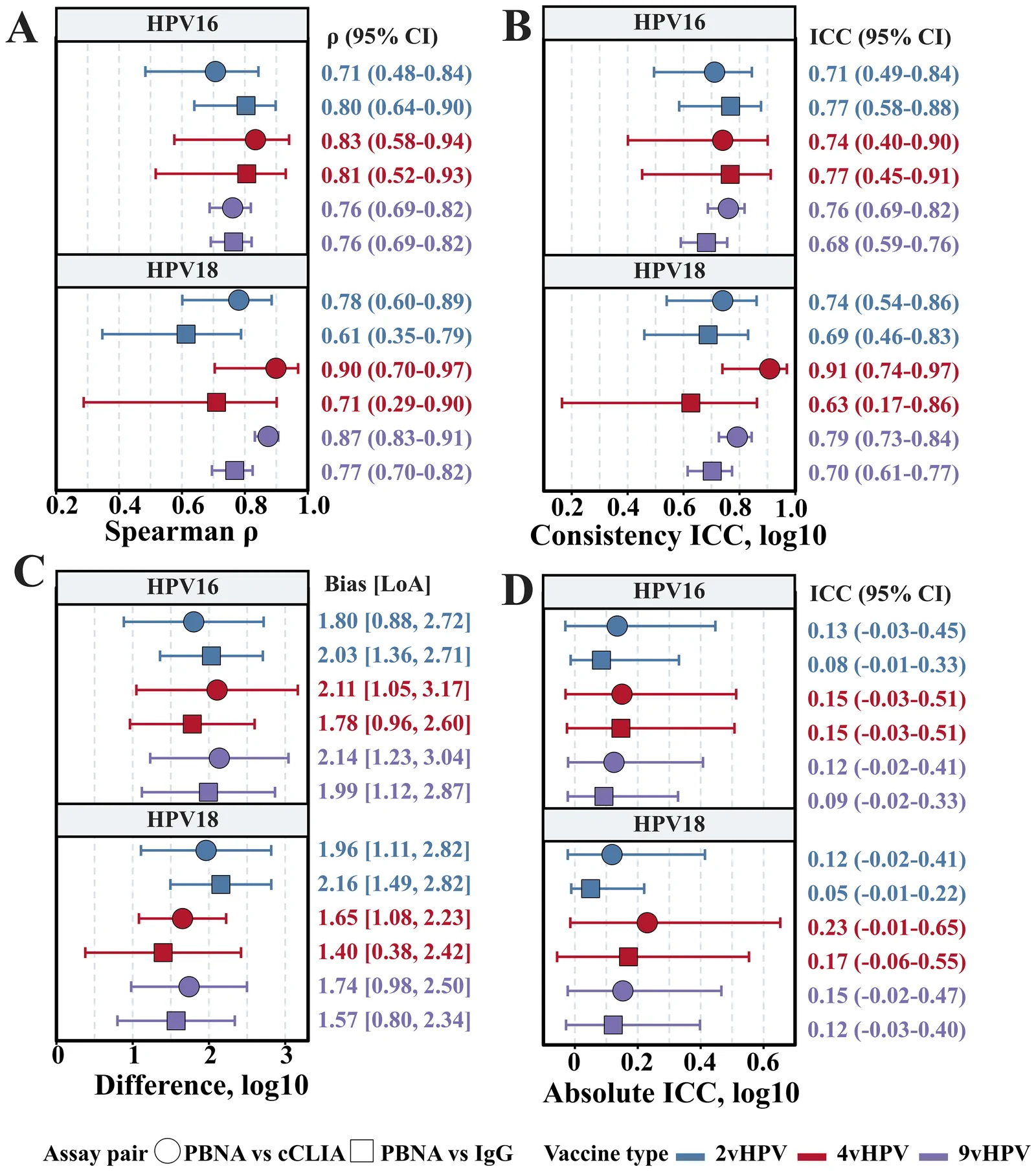
Vaccine-type-stratified relationships between PBNA and high-throughput assays. **(A)** Spearman’s rank correlations between PBNA and cCLIA or IgG for HPV16- and HPV18-specific antibodies across vaccine types. **(B)** Consistency ICCs based on log_10_-transformed assay results. **(C)** Bland–Altman mean bias and 95% limits of agreement based on log_10_-transformed differences. Differences were calculated as PBNA minus cCLIA or PBNA minus IgG. **(D)** Absolute-agreement ICCs based on log_10_-transformed assay results. Circles indicate comparisons between PBNA and cCLIA, and squares indicate comparisons between PBNA and IgG. Colors indicate vaccine type: bivalent, quadrivalent, and nonavalent HPV vaccines. Points represent point estimates. Horizontal bars represent 95% confidence intervals in panels **(A, B, D)**, and 95% limits of agreement in panel **(C)**. PBNA results are expressed as ID_50_, whereas cCLIA and IgG results are expressed in IU/L. PBNA, pseudovirion-based neutralization assay; cCLIA, competitive chemiluminescent immunoassay; IgG, immunoglobulin G; IgG assay, IgG binding antibody assay; ICC, intraclass correlation coefficient; CI, confidence interval; LoA, limits of agreement.

In the cross-sectional study, PBNA was positively associated with cCLIA and IgG among recipients of bivalent, quadrivalent, and nonavalent HPV vaccines (**Figures 5A, B**). This finding indicated that both assays could partly reflect the relative antibody levels measured by PBNA. The relationship between PBNA and cCLIA was generally more stable, especially for HPV18. For HPV18, PBNA and cCLIA showed moderate-to-strong correlations and ICC values for consistency across the three vaccine groups. The relationship between PBNA and IgG was also positive, but the estimates were more variable. This was particularly evident among recipients of bivalent and quadrivalent HPV vaccines, where some confidence intervals were wider.

However, analyses stratified by vaccine type also showed that relative agreement did not imply numerical interchangeability. Bland–Altman analysis showed persistent positive mean bias between PBNA and cCLIA or IgG across vaccine types, with wide 95% limits of agreement (**Figure 5C**). The mean bias ranged from approximately 1.40 to 2.16 log_10_ units. ICC values for absolute agreement remained low across HPV types, vaccine types, and assay pairs. These values ranged from approximately 0.05 to 0.23, and most confidence intervals crossed or approached zero (**Figure 5D**). These results indicated that cCLIA and IgG were more suitable for assessing relative antibody levels than for directly replacing quantitative PBNA results across different vaccine backgrounds. Because the quadrivalent HPV vaccine group had a relatively small sample size, its stratified estimates should be interpreted cautiously.

Product-level sensitivity analyses separating Cecolin and Cervarix showed positive relationships of PBNA with cCLIA and IgG across all four vaccine products, although selected estimates were more variable, particularly for HPV18 PBNA–IgG comparisons (**Supplementary Figures 4, 5**). Given the small sizes of several product-specific subgroups, these findings were considered exploratory.

Because age and time since vaccination differed across vaccine groups, an additional multivariable sensitivity analysis was performed (**Supplementary Table 7**). After simultaneous adjustment for vaccine product, age, and time since the last vaccination, age was not independently associated with any inter-assay difference. Time since vaccination showed a modest association with the HPV16 PBNA–cCLIA difference (β per 100 days = 0.0145, 95% CI: 0.0001–0.0290; *P* = 0.048) and was also associated with the HPV18 PBNA–IgG difference (β per 100 days = −0.0204, 95% CI: −0.0342 to −0.0066; *P* = 0.004). No significant time-related associations were observed for HPV16 PBNA–IgG or HPV18 PBNA–cCLIA. After adjustment, the product-level association was attenuated for HPV16 PBNA–IgG (adjusted *P* = 0.406), whereas product-level heterogeneity remained detectable for HPV16 PBNA–cCLIA, HPV18 PBNA–cCLIA, and HPV18 PBNA–IgG. These findings suggest that vaccination timing may contribute to selected product-level differences but does not fully account for the observed heterogeneity.

### 3.6 Longitudinal antibody dynamics and stage-specific assay relationships

**Figure 6:**
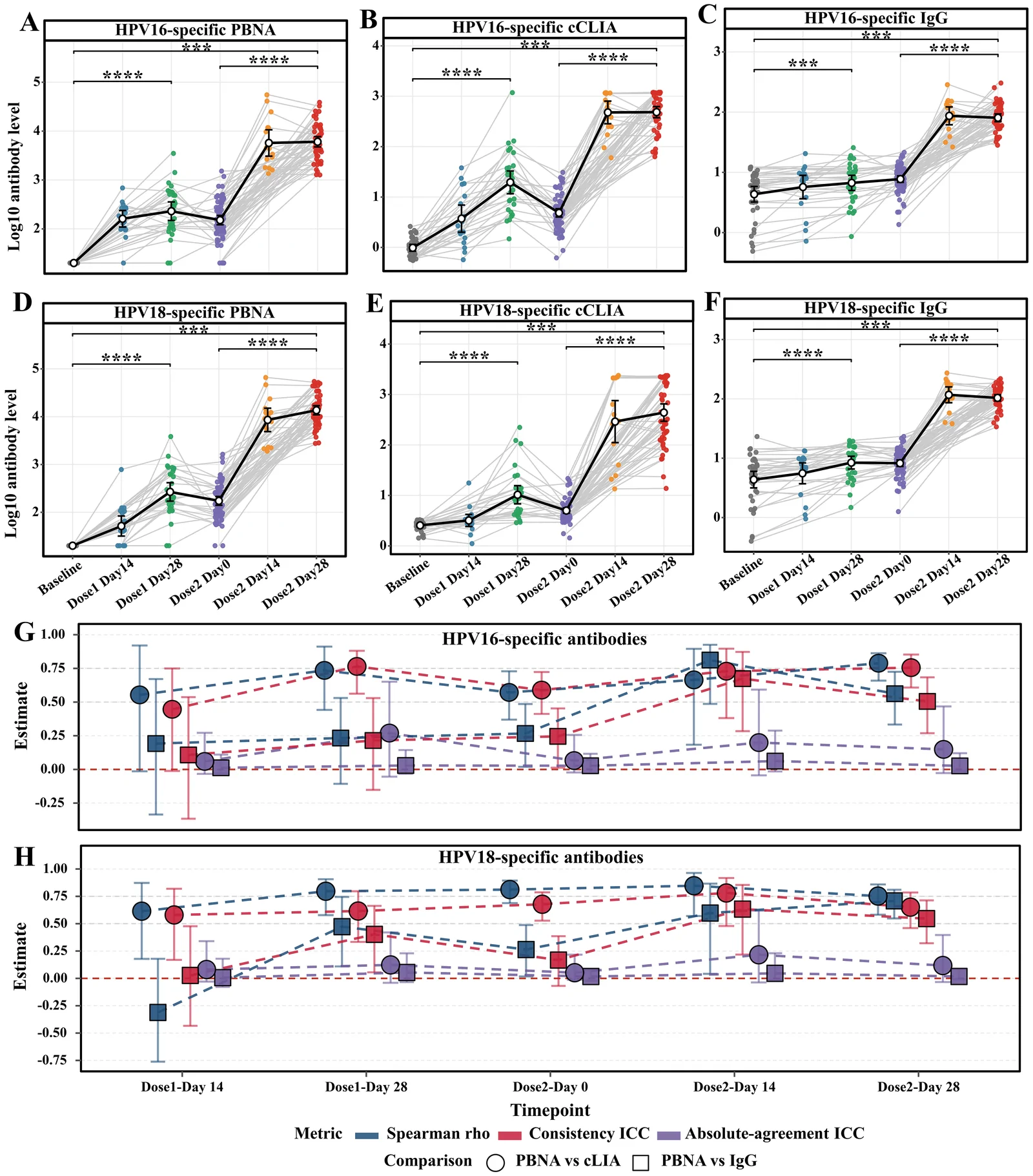
Longitudinal antibody dynamics and time-point-specific assay agreement. **(A)** Longitudinal changes in HPV16-specific PBNA titers across the two-dose vaccination schedule. **(B)** Longitudinal changes in HPV16-specific cCLIA levels. **(C)** Longitudinal changes in HPV16-specific IgG levels. **(D)** Longitudinal changes in HPV18-specific PBNA titers. **(E)** Longitudinal changes in HPV18-specific cCLIA levels. **(F)** Longitudinal changes in HPV18-specific IgG levels. Grey lines represent individual trajectories, colored points represent observed values at each sampling time point, and black points with error bars represent group-level summary estimates. Statistical significance for selected time-point comparisons is indicated above each panel. **(G)** Time-point-specific agreement between PBNA and cCLIA or IgG for HPV16-specific antibodies. **(H)** Time-point-specific agreement between PBNA and cCLIA or IgG for HPV18-specific antibodies. Spearman’s rank correlation coefficients, consistency ICCs, and absolute-agreement ICCs are shown with 95% confidence intervals. Circles indicate comparisons between PBNA and cCLIA, and squares indicate comparisons between PBNA and IgG. Baseline samples were not included in the time-point-specific agreement analysis because antibody levels were close to the lower detection range for most assays. PBNA results are expressed as ID_50_, whereas cCLIA and IgG results are expressed in IU/L. All assay values used for agreement analyses were log_10_-transformed. PBNA, pseudovirion-based neutralization assay; cCLIA, competitive chemiluminescent immunoassay; IgG, immunoglobulin G; IgG assay, IgG binding antibody assay; ICC, intraclass correlation coefficient; CI, confidence interval.

Clear time-dependent changes in HPV16- and HPV18-specific antibody levels were observed after the two-dose HPV vaccination schedule (**Figures 6A–F**). All three assays showed increases in antibody levels after Dose 1 and further increases after Dose 2. However, between Dose 1 Day 28 and Dose 2 Day 0, the magnitude of decline differed across assays: PBNA showed a moderate decrease, cCLIA showed a more pronounced decrease, whereas IgG levels changed relatively little. Antibody levels increased again after Dose 2 and remained elevated at Dose 2 Day 28. Overall, all three methods captured major temporal pattern of vaccine-induced antibody responses, although the magnitude of change during the contraction phase differed across platforms.

Analyses by sampling time point further showed that the relationships of PBNA with cCLIA and IgG changed across vaccination stages (**Figures 6G, H**). Overall, PBNA and cCLIA showed relatively stable correlations and ICC values for consistency across post-vaccination time points, particularly at 28 days after Dose 1 and after Dose 2. In contrast, the relationship between PBNA and IgG fluctuated more clearly. During the early stage after Dose 1, the correlation and ICC for consistency between PBNA and IgG were relatively low, with wider confidence intervals for some estimates. After Dose 2, the relative agreement between PBNA and IgG improved, but it remained less stable than the agreement between PBNA and cCLIA.

Consistent with the findings from the vaccine-type analyses, ICC values for absolute agreement remained low throughout follow-up in the longitudinal cohort (**Figures 6G, H**). For both HPV16 and HPV18, ICC values for absolute agreement between PBNA and cCLIA or IgG did not show clear improvement after Dose 2. These findings suggested that increased antibody levels after vaccination may improve relative agreement between assays, but are insufficient to eliminate absolute numerical differences.

Overall, the stratified analyses showed that cCLIA and IgG could partly reflect relative antibody responses measured by PBNA, but the stability of these relationships varied by vaccine type and vaccination stage. The relationship between PBNA and cCLIA was generally more stable than that between PBNA and IgG, particularly for HPV18 and after Dose 2. Nevertheless, the limited absolute numerical agreement indicated that cCLIA and IgG should not be considered direct substitutes for continuous PBNA values without assay-specific calibration.

## 4 Discussion

This study systematically compared the performance of the PBNA, cCLIA, and VLP-based IgGassay for HPV16- and HPV18-specific antibody assessment in cross-sectional and a real-world longitudinal cohort. All three assays captured the general pattern of antibody responses after HPV vaccination, but their relative agreement, classification performance, and numerical comparability differed across assays and vaccination stages.

The present findings are broadly consistent with previous comparisons of HPV serological assays. In studies of the quadrivalent HPV vaccine Gardasil, the correlation coefficients between the pseudovirus neutralizing antibody assay and the Merck competitive Luminex immunoassay (cLIA) were 0.672 for HPV16 and 0.905 for HPV18 (12). Quang et al. reported high concordance between HPV-specific IgG measured by a multiplex bead-based immunoassay and PBNA-measured neutralizing antibodies (13). Similarly, Panicker et al. showed that a multiplex M9ELISA correlated strongly with PBNA across nine HPV vaccine types (14). Consistent with these reports, PBNA and cCLIA showed generally strong and relatively stable correlations and consistency ICCs in the present study, particularly in the longitudinal cohort, whereas the PBNA–IgG relationship varied more across vaccination stages. However, these measures indicate relative inter-assay agreement rather than numerical equivalence. The low absolute-agreement ICCs and persistent Bland–Altman bias therefore show that strong relative agreement does not support direct quantitative interchangeability. Passing–Bablok regression further showed that explicit assay-specific calibration equations could be derived, but their coefficients varied across HPV types and study populations. These equations should therefore be regarded as exploratory, setting-specific calibration functions rather than universal conversion standards; external validation is required before quantitative application.

The classification and absolute agreement analyses further clarified the application boundaries of the two high-throughput assays (15, 16). In the cross-sectional dataset, both cCLIA and IgG discriminated PBNA-defined serostatus well. Therefore, the ROC analyses evaluate agreement with PBNA-defined serostatus rather than the ability of either assay to predict clinical protection. However, these results should be interpreted in light of the study population. The inclusion of unvaccinated controls increased the number of PBNA-negative samples and may also have widened the separation between positive and negative groups. Therefore, the observed AUC and specificity may appear more favorable than those expected in vaccinated populations, particularly among individuals with antibody levels close to the PBNA-defined threshold. In the longitudinal cohort, cCLIA showed more stable classification performance than IgG. The relatively low sensitivity of the fixed VLP-IgG cut-offs suggests that manufacturer-recommended thresholds may not be optimal for longitudinal monitoring across different post-vaccination stages. Although threshold optimization improved classification performance, data-derived cut-offs require external validation before broader application. Time-point-specific ROC analysis was also limited because PBNA-negative samples became rare or absent after vaccination. Thus, the pooled longitudinal ROC estimates should not be assumed to apply equally across all vaccination stages.

The longitudinal analyses further showed that inter-assay relationships changed across vaccination stages. The PBNA–IgG relationship was less stable during the early response after Dose 1 and improved after Dose 2. The assay-dependent differences observed during the contraction phase between Dose 1 Day 28 and Dose 2 Day 0 further indicate that the three platforms do not capture antibody dynamics identically. The relatively flat VLP-IgG profile may reflect lower sensitivity to dynamic changes during antibody contraction, whereas the greater apparent decline observed with cCLIA relative to PBNA suggests that the magnitude of longitudinal change may depend on the assay platform. These stage-dependent differences are biologically plausible because the three assays capture different antibody dimensions. PBNA measures functional neutralizing activity, competitive assays detect a narrower subset of antibodies that recognize specific neutralization-related epitopes, and VLP-based IgG assays measure a broader spectrum of binding antibodies that may include neutralizing, non-neutralizing, and cross-reactive antibodies (17, 18). Yao et al. reported limited agreement between HPV-specific IgG and neutralizing antibodies in unvaccinated females, particularly when antibody levels were low or when the immune background was not vaccine-induced (19).

One possible explanation is that PBNA is not restricted to a specific immunoglobulin isotype and may capture neutralizing activity contributed by early IgM and IgA responses, whereas the VLP-IgG assay measures only IgG. Accordingly, IgM- or IgA-mediated neutralizing activity during the early post-vaccination period may be detected by PBNA but not by the VLP-IgG assay, which may partly contribute to the lower early sensitivity of IgG (20). Because IgM and IgA were not measured separately in this study, this mechanism remains a possible biological explanation rather than a directly demonstrated finding. After Dose 2, repeated antigen exposure and affinity maturation may enrich higher-affinity IgG antibodies capable of recognizing neutralization-related epitopes, which may partly explain the improved and more stable PBNA-IgG relationship after the second dose (21–23).

Vaccine-specific analyses should also be interpreted in the context of heterogeneous vaccination backgrounds. Vaccine groups differed in age and time since the last dose, and product-level sensitivity analyses showed variation in selected inter-assay relationships. After adjustment for vaccine product, age, and vaccination timing, age was not independently associated with inter-assay differences, whereas time since vaccination was associated with selected comparisons, including HPV16 PBNA–cCLIA and HPV18 PBNA–IgG. Product-level heterogeneity nevertheless remained for several comparisons after adjustment. Therefore, differences in bias or agreement across vaccine products should not be attributed specifically to vaccine valency, adjuvant formulation, or intrinsic assay limitations. Rather, they should be interpreted in the context of vaccination timing and antibody kinetics, vaccine-product background, and assay-specific measurement characteristics. Given the small sizes of several product-specific subgroups, these findings remain exploratory.

Taken together, these findings support a scenario-based testing framework for HPV vaccine immune monitoring. Within this framework, PBNA may serve as a functional laboratory reference for neutralizing activity, cCLIA may support high-throughput serological assessment, and IgG testing may support population-level antibody trend monitoring. The exploratory combined-classification analysis further showed that cCLIA and IgG can be combined according to different priorities, including sensitivity, specificity, and testing burden (23, 24). Importantly, this scenario-based framework remains conceptual and requires prospective validation in independent populations and real-world testing settings before implementation in public health surveillance or clinical laboratories. This study has several limitations. First, the cross-sectional sample included heterogeneous vaccination backgrounds and a relatively small quadrivalent subgroup; although adjusted analyses considered vaccine product, age, and time since vaccination, residual confounding may remain. Second, some longitudinal cohort time points also had limited sample sizes, and repeated observations from the same participants may introduce within-participant dependence. Although cluster bootstrap and subject-mean sensitivity analyses yielded results consistent with the primary analyses, residual dependence cannot be completely excluded. Third, PBNA values below the reporting threshold were not quantitatively resolved; although their exclusion had little effect on rank-based results, selected proportional-bias estimates changed, and PBNA negativity does not establish absence of a vaccine-induced antibody response. Fourth, PBNA may be affected by inter-laboratory variability, and the lack of fully harmonized international standards limits direct quantitative comparison across laboratories and platforms. Finally, the absence of clinical endpoints, such as HPV infection or HPV-related lesions, and the restriction to females from Guangdong Province limit inference about protective antibody thresholds and generalizability to other populations.

## 5 Conclusion

In this real-world study, PBNA, cCLIA, and the IgG binding assay successfully captured HPV16- and HPV18-specific antibody responses, yet demonstrated distinct application boundaries. cCLIA exhibited a relatively stable relationship with PBNA and may support high-throughput serological assessment, whereas the IgG assay may be more suitable for monitoring population-level antibody response patterns. Importantly, exploratory assay-specific calibration equations could be derived, but their variation across HPV types and study settings indicates that they should not yet be regarded as universal substitutes for direct PBNA measurement. Based on these findings, we propose a tailored, scenario-based monitoring strategy: PBNA for reference calibration and mechanistic evaluation, cCLIA for high-throughput serological assessment, IgG for large-scale epidemiological surveillance, and combined cCLIA-IgG interpretation when sensitivity, specificity, or testing burden must be explicitly prioritized. This framework requires prospective validation before implementation in public health surveillance or clinical laboratory settings.

## Data Availability

The raw data supporting the conclusions of this article will be made available by the authors, without undue reservation.
