# Supplementary material for: Assessing high-throughput surrogate assays for real-world HPV16- and HPV18-specific vaccine immune monitoring: a comparison of PBNA, cCLIA, and IgG
Source: Front Immunol. 2026 Sep 8;17:1923707. doi: 10.3389/fimmu.2026.1923707 (PMC13593855; doi:10.3389/fimmu.2026.1923707)
Supplement: Supplementary file 1 [file SupplementaryFile1.docx]

**Supplementary Table***

**Supplementary Table 1A.**

**Diagnostic performance metrics of cCLIA and IgG assays for identifying PBNA-defined seropositivity in the cross-sectional study.**

| **HPV Type** | **Assay** | **Cut-off Strategy** | **TP** | **FN** | **FP** | **TN** | **Sensitivity (% [95% CI])** | **Specificity (% [95% CI])** | **Youden Index** | **Kappa [95% CI]** |
| --- | --- | --- | --- | --- | --- | --- | --- | --- | --- | --- |
| HPV16 | cCLIA | Manufacturer-recommended | 167 | 46 | 4 | 46 | 78.4 [72.3, 83.7] | 92.0 [80.8, 97.8] | 0.704 | 0.533 [0.419, 0.637] |
|  |  | Youden-optimized | 172 | 41 | 4 | 46 | 80.8 [74.8, 85.8] | 92.0 [80.8, 97.8] | 0.728 | 0.567 [0.454, 0.670] |
| HPV16 | IgG | Manufacturer-recommended | 180 | 33 | 4 | 46 | 84.5 [78.9, 89.1] | 92.0 [80.8, 97.8] | 0.765 | 0.626 [0.519, 0.723] |
|  |  | Youden-optimized | 170 | 43 | 0 | 50 | 79.8 [73.8, 85.0] | 100.0 [92.9, 100.0] | 0.798 | 0.601 [0.502, 0.699] |
| HPV18 | cCLIA | Manufacturer-recommended | 172 | 36 | 4 | 51 | 82.7 [76.9, 87.6] | 92.7 [82.4, 98.0] | 0.754 | 0.621 [0.513, 0.722] |
|  |  | Youden-optimized | 167 | 41 | 1 | 54 | 80.3 [74.2, 85.5] | 98.2 [90.3, 100.0] | 0.785 | 0.619 [0.518, 0.710] |
| HPV18 | IgG | Manufacturer-recommended | 182 | 26 | 10 | 45 | 87.5 [82.2, 91.7] | 81.8 [69.1, 90.9] | 0.693 | 0.626 [0.515, 0.729] |
|  |  | Youden-optimized | 177 | 31 | 4 | 51 | 85.1 [79.5, 89.6] | 92.7 [82.4, 98.0] | 0.778 | 0.659 [0.550, 0.750] |

Abbreviations: CI, confidence interval; cCLIA, chemiluminescence immunoassay; FN, false negative; FP, false positive; IgG, immunoglobulin G; PBNA, pseudovirus-based neutralization assay; TN, true negative; TP, true positive.

**Supplementary Table 1B.**

**Diagnostic performance metrics of cCLIA and IgG assays for identifying PBNA-defined seropositivity in the longitudinal cohort.**

| **HPV Type** | **Assay** | **Cut-off Strategy** | **TP** | **FN** | **FP** | **TN** | **Sensitivity (% [95% CI])** | **Specificity (% [95% CI])** | **Youden Index** | **Kappa [95% CI]** |
| --- | --- | --- | --- | --- | --- | --- | --- | --- | --- | --- |
| HPV16 | cCLIA | Manufacturer-recommended | 127 | 52 | 0 | 45 | 70.9 [63.7, 77.5] | 100.0 [92.1, 100.0] | 0.709 | 0.495 [0.387, 0.594] |
|  |  | Youden-optimized | 159 | 20 | 5 | 40 | 88.8 [83.3, 93.0] | 88.9 [75.9, 96.3] | 0.777 | 0.691 [0.568, 0.794] |
| HPV16 | IgG | Manufacturer-recommended | 101 | 78 | 4 | 41 | 56.4 [48.8, 63.8] | 91.1 [78.8, 97.5] | 0.475 | 0.294 [0.204, 0.387] |
|  |  | Youden-optimized | 115 | 64 | 7 | 38 | 64.2 [56.8, 71.3] | 84.4 [70.5, 93.5] | 0.487 | 0.330 [0.222, 0.441] |
| HPV18 | cCLIA | Manufacturer-recommended | 121 | 53 | 0 | 50 | 69.5 [62.1, 76.3] | 100.0 [92.9, 100.0] | 0.695 | 0.505 [0.407, 0.605] |
|  |  | Youden-optimized | 154 | 20 | 1 | 49 | 88.5 [82.8, 92.8] | 98.0 [89.4, 99.9] | 0.865 | 0.762 [0.665, 0.852] |
| HPV18 | IgG | Manufacturer-recommended | 105 | 69 | 8 | 42 | 60.3 [52.7, 67.7] | 84.0 [70.9, 92.8] | 0.443 | 0.309 [0.205, 0.416] |
|  |  | Youden-optimized | 133 | 41 | 14 | 36 | 76.4 [69.4, 82.5] | 72.0 [57.5, 83.8] | 0.484 | 0.406 [0.270, 0.528] |

Abbreviations: CI, confidence interval; CLIA, chemiluminescence immunoassay; FN, false negative; FP, false positive; IgG, immunoglobulin G; PBNA, pseudovirus-based neutralization assay; TN, true negative; TP, true positive.

Supplementary Figure 1. Subject-mean sensitivity analysis of pairwise correlations among PBNA, cCLIA, and IgG assays in the longitudinal cohort. (A) Correlation between HPV16-specific PBNA and cCLIA. (B) Correlation between HPV16-specific PBNA and IgG. (C) Correlation between HPV16-specific cCLIA and IgG. (D) Correlation between HPV18-specific PBNA and cCLIA. (E) Correlation between HPV18-specific PBNA and IgG. (F) Correlation between HPV18-specific cCLIA and IgG. Each point represents one participant-level mean value calculated from log10-transformed assay results across available longitudinal time points. Spearman’s rank correlation coefficients and P values were recalculated using participant-level mean values. Fitted lines are shown for trend visualization only. PBNA titers are expressed as ID50 values, whereas cCLIA and IgG values are expressed in IU/L. Abbreviations: cCLIA, competitive chemiluminescent immunoassay; IgG, immunoglobulin G; PBNA, pseudovirion-based neutralization assay. Supplementary Figure 2. Subject-mean Bland–Altman analyses of agreement among PBNA, cCLIA, and IgG assays in the longitudinal cohort. (A) Bland–Altman plot for HPV16-specific PBNA versus cCLIA. (B) Bland–Altman plot for HPV16-specific PBNA versus IgG. (C) Bland–Altman plot for HPV16-specific cCLIA versus IgG. (D) Bland–Altman plot for HPV18-specific PBNA versus cCLIA. (E) Bland–Altman plot for HPV18-specific PBNA versus IgG. (F) Bland–Altman plot for HPV18-specific cCLIA versus IgG. Each point represents one participant-level mean value calculated from log10-transformed assay results across available longitudinal time points. The x-axis represents the mean of two log10-transformed assay values, and the y-axis represents their difference. Difference directions are defined as PBNA minus cCLIA, PBNA minus IgG, or cCLIA minus IgG. Solid horizontal lines indicate mean bias, and dashed horizontal lines indicate the 95% limits of agreement. PBNA titers are expressed as ID50 values, whereas cCLIA and IgG values are expressed in IU/L.

**Supplementary Table 2A.**

**Characteristics of vaccinated participants in the cross-sectional study by vaccine type.**

| **Characteristic** | **Overall** | **Bivalent** | **Quadrivalent** | **Nonavalent** | ***P*** |
| --- | --- | --- | --- | --- | --- |
| Age, years, median (IQR) | 25.0  (24.0-26.0) | 25.0  (23.0-27.0) | 27.5  (25.0-29.5) | 25.0  (24.0-26.0) | 0.009 |
| Height, cm, median (IQR) | 162.0  (158.0-166.0) | 163.0  (159.0-168.0) | 161.0  (158.0-164.5) | 162.0  (158.0-166.0) | 0.437 |
| BMI, kg/m², median (IQR) | 20.26  (19.03-21.56) | 20.20  (18.59-21.97) | 20.87  (18.81-24.02) | 20.20  (19.05-21.48) | 0.507 |
| Time since last dose, days, median (IQR) | 336  (205-754) | 411  (222-718) | 948  (503-1426) | 326  (183-686) | 0.007 |

**Supplementary Table 2B.**

**Characteristics of participants in the longitudinal cohort by sampling time point.**

| **Characteristic** | **Baseline** | **Dose1 Day14** | **Dose1 Day28** | **Dose2 Day0** | **Dose2 Day14** | **Dose2 Day28** | ***P*** |
| --- | --- | --- | --- | --- | --- | --- | --- |
| Age, years, median (IQR) | 12.0  (12.0-13.0) | 12.0  (12.0-13.0) | 12.0  (12.0-13.0) | 13.0  (12.0-13.0) | 13.0  (13.0-13.5) | 13.0  (12.0-13.0) | 1.000 |
| Height, cm, median (IQR) | 157.5  (153.0-160.0) | 158.0  (153.0-161.0) | 159.0  (153.0-161.0) | 158.0  (155.0-163.0) | 156.0 (154.0-162.0) | 160.0  (155.0-163.0) | 0.400 |
| BMI, kg/m², median (IQR) | 18.6  (17.1-20.2) | 19.0  (17.3-20.3) | 18.6  (17.1-20.0) | 18.8  (17.5-20.7) | 20.6  (18.2-23.0) | 18.7  (17.3-20.4) | 0.401 |
